# GRIN: “GRoup versus INdividual physiotherapy following lower limb intra-muscular Botulinum Toxin-A injections for ambulant children with cerebral palsy: an assessor-masked randomised comparison trial”: study protocol

**DOI:** 10.1186/1471-2431-14-35

**Published:** 2014-02-07

**Authors:** Rachel E Thomas, Leanne M Johnston, Roslyn N Boyd, Leanne Sakzewski, Megan J Kentish

**Affiliations:** 1 Queensland Cerebral Palsy Health Service, The Royal Children’s Hospital, Brisbane, Australia; 2 Cerebral Palsy League, Brisbane, Australia; 3 School of Health and Rehabilitation Sciences, The University of Queensland, Brisbane, Australia; 4 Queensland Cerebral Palsy and Rehabilitation Research Centre, School of Medicine, The University of Queensland, Brisbane, Australia

**Keywords:** Botulinum Toxin-A, Cerebral palsy, Group physiotherapy, Models of training, Physiotherapy, Rehabilitation, Assessor-masked randomised comparison trial

## Abstract

**Background:**

Cerebral palsy is the most common cause of physical disability in childhood. Spasticity is a significant contributor to the secondary impairments impacting functional performance and participation. The most common lower limb spasticity management is focal intramuscular injections of Botulinum Toxin-Type A accompanied by individually-delivered (one on one) physiotherapy rehabilitation. With increasing emphasis on improving goal-directed functional activity and participation within a family-centred framework, it is timely to explore whether physiotherapy provided in a group could achieve comparable outcomes, encouraging providers to offer flexible models of physiotherapy delivery. This study aims to compare individual to group-based physiotherapy following intramuscular Botulinum Toxin-A injections to the lower limbs for ambulant children with cerebral palsy aged four to fourteen years.

**Methods/Design:**

An assessor-masked, block randomised comparison trial will be conducted with random allocation to either group-based or individual physiotherapy. A sample size of 30 (15 in each study arm) will be recruited. Both groups will receive six hours of direct therapy following Botulinum Toxin-A injections in either an individual or group format with additional home programme activities (three exercises to be performed three times a week). Study groups will be compared at baseline (T1), then at 10 weeks (T2, efficacy) and 26 weeks (T3, retention) post Botulinum Toxin-A injections. Primary outcomes will be caregiver/s perception of and satisfaction with their child’s occupational performance goals (Canadian Occupational Performance Measure) and quality of gait (Edinburgh Visual Gait Score) with a range of secondary outcomes across domains of the International Classification of Disability, Functioning and Health.

**Discussion:**

This paper outlines the study protocol including theoretical basis, study hypotheses and outcome measures for this assessor-masked, randomised comparison trial comparing group versus individual models of physiotherapy following intramuscular injections of Botulinum Toxin-A to the lower limbs for ambulant children with cerebral palsy.

**Trial registration:**

ACTRN12611000454976

## Background

Cerebral palsy (CP) is the most common cause of physical disability in childhood with an incidence of 2.11 per 1000 live births [1]. It describes a “group of permanent disorders of the development of movement and posture, causing activity limitations that are attributed to non-progressive disturbances that occurred in the developing fetal or infant brain” [2]. In 2013, the Australian Cerebral Palsy Register reported that 71% of children with CP achieved ambulation and 91% were classified with spasticity as the predominant motor type. Children with lower limb spasticity often experience a range of impairments including weakness, tightness, reduced motor control and muscle selectivity. These impairments can lead to limitations in functional ability, balance, ambulation and fitness compared to typically developing peers [3,4]. Physiotherapy intervention focuses on reducing these impairments and optimising functional, goal-related performance.

Focal, intramuscular Botulinum Toxin-Type A (BoNT-A) injections to the lower limb are commonly used in combination with physiotherapy as the temporary reduction in spasticity provides an opportunity to facilitate rehabilitation outcomes [5-8]. For ambulant children with CP (Gross Motor Functional Classification System, GMFCS-E&R, Levels I-III [9]), there is strong evidence that injections of BoNT-A are safe and reduce muscle tone in spastic, active and non-fibrotic lower limb muscles for approximately 12–16 weeks [6,7,10-12]. BoNT-A impacts body structure and function, however the accompanying physiotherapy often targets activity level outcomes (International Classification of Functioning, Disability and Health, ICF [13]).

Functional improvement in ambulant children with CP has been reported in a number of randomised controlled trials (RCTs) comparing intramuscular lower limb BoNT-A injections with rehabilitation (physiotherapy, casting and/or orthotic management), to a control group of rehabilitation alone with or without placebo injections. Rehabilitation combined with BoNT-A injections demonstrated significantly greater improvement in gross motor function as measured by the Gross Motor Function Measure (GMFM-88 or-66) [14-18] and quality of gait using the Physician’s Rating Scale [14,19-22], Edinburgh Visual Gait Score (EVGS) [23] or Three Dimensional Gait Analysis (3DGA) [20]. Improvement in performance-related goals have been reported when measured by the Canadian Occupational Performance Measure (COPM) [17], Goal Attainment Scaling (GAS) [24] or parental questionnaires [15,16,25].

Despite the acknowledged success of physiotherapy rehabilitation combined with BoNT-A injections, relative effectiveness of the specific components of physiotherapy rehabilitation, including intensity and dose, is difficult to interpret because it is often poorly described [14,15,17,25]. Results of a number of systematic reviews and consensus papers found limited evidence to support or refute individual physiotherapy modalities post lower limb BoNT-A injections [5,7,11,26]. The content of physiotherapy rehabilitation outlined in reported studies has included: active and passive stretching of muscle agonists [16,18,22,23,27]; functional or resistive strengthening of the antagonists [16,18,22,23,27]; functional mobility training and/or gait training [16,18,23,27]. One retrospective, controlled intervention study has directly compared the specific content of two physiotherapy approaches following lower limb BoNT-A injections [28]. Thirty-eight children with CP (mean age 7 years, 7 months, GMFCS I-III, 11 unilateral, 27 bilateral motor distribution) who received Neurodevelopmental Treatment (NDT, mean total dose 24.2 hours) were randomly selected and retrospectively matched to a group of children who received conventional physiotherapy (CPT, mean total dose 20.5 hours). Content of physiotherapy, determined via therapist questionnaires, and improvement of impairment and gait-related goals (GAS) were compared between groups two months post injection. Both approaches utilized muscle tone inhibition techniques, stretching, strengthening and functional training, with the NDT group spending a greater proportion of time on functional training (NDT 42%; CPT 28%, *p* = 0.009). Whilst the NDT group showed greater goal attainment post intervention (mean converted GAS score NDT 56, CPT 52, *p* = 0.008), results should be interpreted cautiously. Therapy content was only described and analysed for 62% (n = 47) of children due to reduced completion of questionnaires by treating physiotherapists. As GAS goals were impairment-based it is unclear from this study if a more functional approach to training translates to improvement in goals related to function and participation.

In the absence of high level evidence, expert opinion and consensus statements recommend post BoNT-A physiotherapy includes functional and targeted motor training in combination with serial casting, stretching and strengthening [7,8,29]. Additionally, intervention should incorporate: (1) collaborative, individualized, realistic and specific goal setting which span across all domains of the ICF [7,29]; (2) specificity of task and training [30,31]; (3) repetition and practise within a functional “just right” context [32,33]; (4) environmental adaption [34] and (5) strategies to increase motivation and engagement [35,36]. This approach will facilitate rehabilitation focused on each child’s specific goals and functional needs [6,33,37]. Studies investigating the efficacy of physiotherapy combined with BoNT-A injections have consistently included therapy delivered in an individual model. It is unclear whether similar outcomes could be achieved using alternative methods of physiotherapy delivery.

Four models of therapy delivery have been reported in the literature for children with CP including: (1) individual (one on one); (2) group-based (three or more participants with similar abilities [38]); (3) web-based training or virtual reality [39,40]; (4) individual consultation with intervention performed as a home programme [41]. Group-based training has been shown to achieve positive rehabilitation outcomes through maximising engagement, motivation and participation [36,42]. Relative effectiveness of group versus individual physiotherapy post lower limb BoNT-A injections has not been examined to date, however. Independent of BoNT-A, effectiveness of group versus standard individualised care has been compared for ambulant children with CP receiving strength, endurance and fitness training [43,44], progressive functional strength training [45,46] and goal-directed activity-focused physiotherapy [47]. Results are difficult to compare due to the heterogeneity of theoretical focus, therapy dose and outcome measures used. However, these studies provide useful guidelines for the elements that contribute to successful group-based physiotherapy.

Effective group-based physiotherapy interventions report similar session structure including warm up, specific intervention activities and then warm down. To maintain motivation and specificity of practise, a combination of group-based activity and individual or paired circuit activity has been recommended [43,44,46,47]. To ensure adequate supervision and progression of exercises, group sizes have been limited to small (4–6 children) [44,45,47] or medium (7–9 children) [43]. Effective dose is not well understood due to variability in intensity, frequency and duration of intervention. Total direct therapy dose has varied from 36 hours [43,45] to 70 hours [44], delivered in varying intensity from three week blocks (intensive model) [47] to 34.6 weeks (distributed model) [44]. Individual session duration has ranged from 45 to 180 minutes with a frequency of two to five sessions per week. Indirect treatment dose achieved via home programme is difficult to interpret due to inadequate reporting. Despite insufficient evidence to confirm optimal group format, several studies found that group-based therapy can achieve equal or greater improvement in outcomes across ICF domains when compared to individual standard care. These include improvements in gross motor ability (GMFM-66) [43,44,47], crouch gait (3DGA) [48], participation (Children’s Assessment of Participation and Enjoyment: CAPE) [44], health-related quality of life (TACQOL) [44] and goal attainment (GAS) [47]. One RCT (n = 51) compared group-based progressive resistance exercise strength training to individualised standard care in 51 ambulant children with CP (GMFCS I-III; mean age 10y 5mo, SD 1y 10mo; 29 male) [45,46]. This study reported no improvement in either group for gross motor ability (GMFM-66), walking ability (One Minute Fast Walk Test) or participation (CAPE). No studies have reported inferior outcomes for group-based interventions compared to individual standard care. Additional qualitative benefits of a group-based model have been reported in the context of the self-determination theory [49,50]. A group program may enhance self-regulation and engagement in the therapy process by attending to children’s basic psychological needs for Autonomy, Relatedness and Competence. There is potential for greater promotion of autonomy by allowing choice (personal goal setting), and through enjoyment, having fun and behaviour modelling to master activities [36,51]. A sense of competence may be fulfilled through scaffolding activities to promote skill development, and providing opportunities for peer learning [52-54] and healthy competition [30,36]. Social support and working with children with similar needs may also increase the feeling of relatedness [36,55].

A group model has the potential to meet the physiotherapy rehabilitation needs for children post lower limb BoNT-A injections. To date there has been no study that has directly compared dose and content-matched group versus individual models of functional, goal-directed physiotherapy rehabilitation following lower limb BoNT-A injections for ambulant children with CP. This study aims to compare the efficacy of these two models to enable informed choice of post BoNT-A physiotherapy rehabilitation delivery.

## Methods/Design

### Study aims

This assessor-masked, parallel group, block RCT aims to compare group versus individual models of physiotherapy following intramuscular lower limb injections of BoNT-A for ambulant school-aged children with CP in the ICF domains of impairment (quality of gait, functional reach), activity/participation (caregiver/s perception of and satisfaction with their child’s occupational performance, gross motor function, walking efficiency) and quality of life [13]. A secondary aim is to gain qualitative feedback from treating physiotherapists, caregivers and participants involved in the study to determine acceptability of the two treatment models.

Study hypotheses are based on the reported benefits of a group model being at a participation and contextual level, so outcomes reflecting a child’s occupational performance, participation or quality of life may improve more in the group-based intervention. Individual training has the potential for greater intensity, specificity of training and repetition of practise of skills. Outcomes at the body, structure and function or activity level [13] could improve more with individual training. Consequently, the specific hypotheses to be tested are:

### Primary hypotheses

**H1:** Compared to individual physiotherapy, group physiotherapy will result in greater improvement in caregivers’ perception of performance of and satisfaction with their child’s occupational goal areas (COPM). This will be observed as between group differences of two or more COPM performance and satisfaction points at T2 (10 weeks-efficacy) and T3 (26 weeks-retention) [56].

**H2**: Compared to group physiotherapy, individual physiotherapy will result in greater improvement in quality of gait (mean score change of ≥ 4.0 points on the Edinburgh Visual Gait Score) at T2 (10 weeks-efficacy) and T3 (26 weeks-retention) [57-59].

### Secondary hypotheses

**H3:** Compared to group physiotherapy, individual physiotherapy will result in greater improvement in efficiency of gait (One Minute Fast Walk Test [60]), gross motor performance (Gross Motor Function Measure-88 Items D&E [61,62]) and functional balance (forward component of the Pediatric Reach Test [63]) at T2 (10 weeks- efficacy) and T3 (26 weeks- retention).

**H4:** Compared to individual physiotherapy, group physiotherapy will result in greater improvement in quality of life (Cerebral Palsy Quality of Life Questionnaire). This will be observed as a between group difference of five points or more in each domain at T2 (10 weeks- efficacy) and T3 (26 weeks- retention) [64-67].

### Study sample and recruitment

#### Inclusion criteria

The study will include children who:

1. Are aged 4–14 years at study entry;

2. Have a confirmed diagnosis of CP with a predominant motor type of spasticity;

3. Are ambulant (classified GMFCS-E&R Level I-III);

4. Are patients of the Queensland Cerebral Palsy Health Service (CP Health) BoNT-A program;

5. Require lower limb BoNT-A for management of spasticity interfering with lower limb functional goals;

6. Can commit to six weekly sessions of post-BoNT-A physiotherapy in either a group or individual format.

#### Exclusion criteria

Children will be excluded from the study if they:

1. Are unable to complete baseline assessments;

2. Have had orthopaedic or neurological surgery and/or other new spasticity management (e.g. Baclofen) within six months prior to commencing the study;

3. Have intellectual or behavioural difficulties which would limit their ability to participate in the assessment or therapy protocols;

4. Have medical co-morbidities which prevent them from exercising safely (e.g. cardiac or respiratory instability, uncontrolled seizures).

### Criteria for withdrawal/failure to proceed

Children will be classified as failure to proceed if they do not attend a minimum of four hours of direct therapy (<66%), or if they need to be withdrawn from the study due to significant post-injection or other medical complications requiring deviation from the rehabilitation pathway.

### Recruitment

#### Sample size

According to CONSORT guidelines, the sample size calculation is based on adequate power for comparison between the functional effects of group-based and individual physiotherapy intervention at 10 weeks (T2, immediately post intervention). A change score difference of two or more points between groups on the performance scale of the COPM (primary outcome measure) would be considered clinically meaningful [56]. A previous study of lower limb intramuscular injections of BoNT-A yielded a standard deviation of changes of 1.4 and 1.7 for COPM performance [17]. Based on a mean change of two points on the COPM performance scale and a standard deviation of 1.6 points for both groups, significance (alpha) level of 0.05 and 80% power, we require 24 participants (12 in each group). Allowing for 20% drop out, a total of 30 participants (15 in each group) will be recruited.

#### Randomisation

Eligible children will be recruited prospectively from the Queensland CP Health Service (Brisbane, Australia) BoNT-A injecting lists in blocks of four to eight children. For each recruitment period, participants will be evenly distributed into one of two geographical clusters according to residential address. Each cluster will be allocated to group or individual therapy by concealed random allocation. The random sequence will be created via coin flipping by an off-site, non-study researcher masked to all other study data. The outcome (e.g. 1 = group; 2 = individual) will be written on a piece of paper and concealed inside a sequentially numbered envelope and securely stored off-site. At each recruitment period the study coordinator will contact the off-site researcher to open the next consecutive envelope to reveal treatment allocation. At the end of each recruitment period, there will be close to equal numbers of children in each study arm. This process will continue until 30 participants are recruited.

### Therapy protocols and delivery

**Figure 1:**
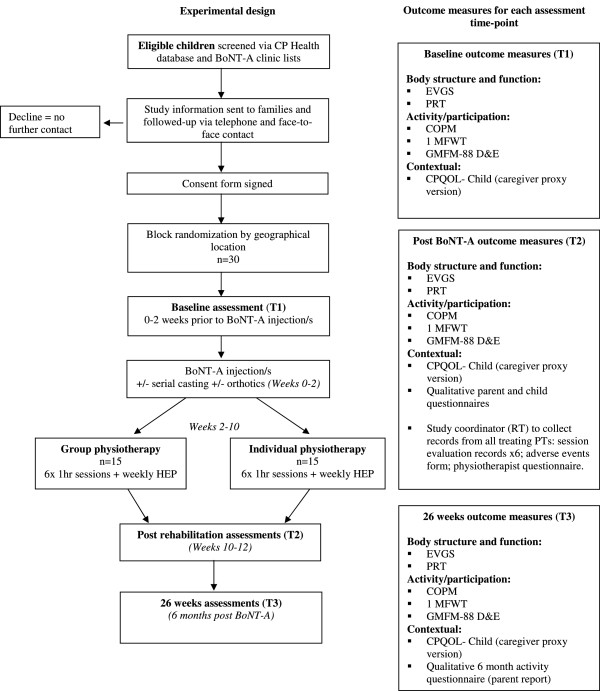
**GRIN flow chart according to CONSORT guidelines.***Legend:* CP Health: Queensland Cerebral Palsy Health Service; BoNT-A: Botulinum Toxin- Type A; EVGS: Edinburgh Visual Gait Scale; PRT: Pediatric Reach Test; COPM: Canadian Occupational Performance Measure; 1MFWT: 1 Minute Fast Walk Test; GMFM-88 D&E: Gross Motor Function Measure-88, Items D&E; CPQOL-Child: Cerebral Palsy Quality of Life Questionnaire; PTs: Physiotherapists; HEP: Home Exercise Programme.

Refer to Figure 1 for the study flow diagram according to CONSORT guidelines.

#### Stage 1: Botulinum Toxin-A injection/s

In accordance with current standard clinical practice, the rehabilitation consultant and physiotherapist will determine muscles to be injected with BoNT-A, following clinical assessment and consultation with the child and their caregiver/s. Muscle selection will vary between participants according to spasticity-related functional impairment and goals of the child and family, as well as from clinical assessment of: gait characteristics (observational gait analysis); resistance to passive stretch (Modified Ashworth Scale [68]); spasticity and dynamic range of movement (Modified Tardieu Scale [69]) and passive range of movement (goniometric and angle finder measures [70]). Dose of BoNT-A will be prescribed according to the Consensus Opinion of the ‘We Move’ Spasticity Study Group (2002), up to a maximum dose of 16 U/kg/body weight, or to a total maximum dose of 400 Units of Botox® (Allergan PLC). Children will receive injections either in theatre under general anaesthetic (100U Botox® diluted in 2 mls of normal saline), or as an outpatient using EMLA cream at the injection site/s (100 U Botox® diluted in 1 ml of normal saline).

#### Stage 2: Serial casting and orthotic prescription

Serial casting will be provided within the three weeks following BoNT-A injections to children with a component of fixed equinus contracture (less than neutral/0 degrees of passive range of ankle dorsiflexion with the knee extended) [7]. Casting will be performed until at least neutral dorsiflexion is achieved. Dose comparisons between study arms will be analysed retrospectively from patient records. Orthoses will be prescribed or modified for each participant by an orthotist as clinically indicated. Moulding for orthoses will occur immediately post BoNT-A for children not requiring casting, and on the day of the last cast fitting for those receiving casting. All orthoses will be available prior to commencement of the rehabilitation block.

#### Stage 3: Physiotherapy intervention

Direct therapy dose for both group and individual models of physiotherapy will be one 60 minute session per week for six weeks (total dose six hours), which is consistent with current funded clinical practice in Queensland. Indirect therapy dose will involve each participant performing three additional individualised, goal-directed activities at home three times a week. Participants will be asked to abstain from any other interventions (e.g. occupational therapy, aquatic therapy) during the rehabilitation phase but can return to their usual therapy following the intervention program. Any therapy or additional structured activity completed during the study will be recorded via a custom-designed questionnaire.

##### Individual therapy

Participants in the individual arm of the study will receive physiotherapy on a one-to-one basis from their usual physiotherapist, who may be located in either a hospital or community out-patient setting.

##### Group therapy

Each group will include at least three participants [38]. If there are insufficient participants to form a group for any recruitment block, additional children with CP (not involved in the study) will be invited to participate. Each additional child will need to meet all study inclusion criteria except they will not have received recent lower limb BoNT-A injections. As for individual rehabilitation, group rehabilitation will be provided in hospital or community settings at a location mutually convenient for each geographical cohort. Group sessions will be directed by one lead physiotherapist with assistance from additional physiotherapist/s, allied health assistant/s or physiotherapy student/s to achieve a minimum ratio of one therapist to three participants. Group dynamics will be encouraged in a variety of ways. For example, children will decide on a name for their group, agree on group rules, receive a team “t-shirt” to wear during sessions and be involved in choosing a theme for the group each week. Activities such as warm up, warm down, stretching and circuit stations will include a combination of group or paired formats. The overall ratio will include a minimum of 60% exercises performed as a group or paired activities and 40% exercises performed simultaneously in a circuit format.

### Content of physiotherapy intervention

Content of the physiotherapy protocol is based on a review of the literature and a clinical practice audit of post BoNT-A rehabilitation providers in Queensland. Exercises have been chosen that require minimal specialised equipment to enable consistent delivery of the program across hospital and community settings. To ensure session structure is the same across both physiotherapy models, a program of suitable exercises and progressions will be provided to treating physiotherapists. Therapists will select and modify exercises to suit each child’s age, intrinsic motivators, individual goals and progression rate. An outline of the session format is displayed in Table 1.

**Table 1:** Example of group and individual physiotherapy session content

**Physiotherapy component**	**Time (minutes)**	**Theoretical rationale**	**Activity examples**
**Warm up activities**	5	Activities have been chosen to prepare the child’s mind and body for the therapy session. In the group model, dynamics will be encouraged by performing games/activities as a group.	• Animal and yoga postures
			• Dancing to music/musical statues
			• Balloon tennis
**Lower limb flexibility**	10	Exercises have been selected to mobilize lower limb joints and muscles through the available range using sustained stretching of agonists injected with BoNT-A for a minimum of five repetitions of 30 seconds duration [71,72].	• In paired long sitting facing each other with feet together, pass the ball between each other (hamstring stretch)
			• Heel dips off the edge of a step (calf stretch)
**Circuit Stations:**		Four stations with one minute rest between each one:	
*Station 1***Functional strengthening**	5	Repetitive, weight-bearing activities have been chosen to improve muscle strength required for functional activities that reflect goals identified via the COPM [36,44]. The focus is on concentric and eccentric muscle activity using body weight resistance. Intensity will be specific to each individual, aiming for 70% of maximum effort, with progression occurring through increasing the number of repetitions or the difficulty of the task (e.g. lower the height of the chair for sit to stand; increase the height of the step for step-ups; increase the speed to complete the activity). Three sets of 10 repetitions is the aim for all participants [73-75].	• Sit to stand
			• Squat to stand
			• Forward and lateral step ups and downs
			• Stair climbing.
*Station 2***Standing and dynamic balance**	5	Activities have been selected to improve the limits of stability in standing tasks relevant to balance goals identified on the COPM.	• Activities in standing where the participant has to reach or squat for an object outside the base of their support
			• Games standing on one leg (SLS) with/without support as required (e.g. dribble a ball around the weight-bearing leg)
*Station 3***Targeted motor control**	5	Individualised activities will be set for each participant to facilitate task practise of functional goals identified on the COPM. Repetition/practise and incremental progression of tasks will occur each week, and through the home programme, to reinforce motor learning [76].	• SLS activities +/− support e.g. dribble ball around standing foot.
			• Kicking to goal (start with large goal area and gradually decrease size).
		*Goal example*:	• Kick ball between 2 people (start with larger ball, decreasing to age appropriate size; progress to kicking ball further to side and increase speed).
		Improve XX ability to kick the ball when playing soccer with friends at school	
		• Be able to make contact with the ball 8 out of 10 attempts	
		• Improve accuracy of kicking to a target (5/10 successful attempts)	
			• Dribble ball around obstacles (e.g. figure of 8 around cones).
*Station 4***Fitness/agility**	5	Activities or games will be carried out to challenge and improve participants’ agility and fitness, aiming for carry-over into physical activity goals (such as being able to run in the playground during school breaks without needing to sit down to rest).	• Timed obstacle races
			• Shuttle runs
			• Relay races.
**Warm down activities**	5	Children will participate in activities that continue to mobilise muscles through range to maximise flexibility, prevent muscle soreness and injury, as well as facilitate reduction in heart rate and temperature to ensure they are in a less aroused state prior to leaving therapy.	• Yoga
			• ‘Simon Says’
			• Songs with actions
**Review of home programme**	10	Review of home programme with each caregiver and participant.	Includes incrementally progressing activities related to the functional performance goals (COPM).

*Key:* BoNT-A: Botulinum Toxin-Type A; COPM: Canadian Occupational Performance Measure; SLS: single leg stance.

### Home exercise programme (HEP)

One key researcher (RT) will design an individualised HEP for each participant. Activities will include part or full task practise of each participant’s key functional goals as identified from the COPM. After the first week, the treating physiotherapist will incrementally progress the HEP activities in liaison with the key researcher as required. Caregivers and/or children will be asked to record the dates, duration and number of repetitions of each activity performed as a HEP, as well as involvement in concurrent activities and/or therapy.

### Treatment fidelity

Physiotherapists providing intervention (group and individual) will be masked to baseline outcome assessments. They will require a minimum of two years’ experience in providing post BoNT-A rehabilitation for children with CP in association with CP Health. In addition, before and during the study, each therapist (and assistants as relevant) will receive face to face or telephone education from the study coordinator (RT) regarding intervention format, content, roles of different staff, exercise and HEP progression. At the start of each rehabilitation block, treating physiotherapists will be provided with standard information for each relevant participant, including the BoNT-A injection date, dose and site/s, baseline musculoskeletal assessment and goals (without score) identified through the COPM. A copy of the physiotherapy protocol will also be provided with suggestions of activities to include in the targeted motor control station, based on each relevant participant/s individualised goals. Physiotherapists will complete a session evaluation form for every participant after each of the six sessions. This includes a summary of activities completed including proportion of time taken on each task. Independent content analysis will determine compliance with the protocol across both arms of the study.

### Outcome measures and procedures

1. **Classification of the sample**

Participants entered into the study will be classified according to:

a) **Gross Motor Functional Classification System** (GMFCS-E&R: [9]):

The GMFCS-E&R is an internationally recognized classification scale for gross motor abilities in children with CP aged two to 18 years ranging from Level I (able to walk independently with limitations in higher gross motor skills) to Level V (unable to sit alone). All children in this study will be ambulant, classified as Levels I-III.

b) **Classification of cerebral palsy:**

Participants will be classified according to motor type/s (primary spasticity), number of limbs involved and unilateral or bilateral distribution [77,78].

c) **Functional mobility:***Functional Mobility Scale (FMS)*

The FMS was designed for children with CP aged four to eighteen years and rates assistive devices required and walking ability at five, 50 and 500 metres which correlate with the child’s ability in the home, school and community settings. The scale ranges from N = does not apply; C = crawling; 1 = uses wheelchair; 2 = uses walker or frame; 3 = uses crutches; 4 = uses sticks (one or two); 5 = independent on level surfaces; 6 = independent on all surfaces [79].

2. **Outcome measures**

Outcome measures will be administered by one physiotherapist (MK) trained and experienced in performing all assessments and one Allied Health Assistant (One Minute Fast Walk Test only). Both assessors will be masked to treatment allocation and previous assessment data. Assessments will be performed at baseline (T1: 0–2 weeks pre BoNT-A injection/s), 10–12 weeks post BoNT-A (T2: on completion of the six week rehabilitation block, efficacy) and at 26 weeks post BoNT-A (T3) to determine medium-term retention of effects. Assessment timeframes are depicted in Figure 1*.* A range of outcome measures will be used across domains of the ICF [13].

2.1 **Body functions and structures**

2.1.1 **Quality of gait:***Edinburgh Visual Gait Score for Cerebral Palsy (EVGS)*

Competence of motor control during gait will be compared to normal values using the EVGS and will be a primary outcome measure for this study. The EVGS is a tabulated scoring system that records 17 joint angles or movements of the trunk and lower limbs during a representative stride. EVGS total score ranges from 0 (best) to 68 (worst) [57]. The EVGS has excellent criterion validity (64% agreement with instrumented gait analysis), repeatability (least significant difference = 3.20 points) and sensitivity to change following surgical intervention (minimal clinically important change: mean score reduction of 4.2 points, range +0.3-8.5) [57-59]. It has good intra-observer reliability which is higher with more experienced observers [23,57,80,81]. Split screen gait analysis will be videotaped simultaneously in the sagittal and frontal planes at the Queensland Children’s Gait Laboratory (QCGL) and independently scored off-site by one physiotherapist (LJ) experienced in EVGS scoring and masked to group allocation, order of assessment and previous data.

2.1.2 **Functional balance***The Pediatric Reach Test (PRT)*

To evaluate the limits of stability in free standing, the forward component of the PRT will be used [63]. In this test, the participant stands with their feet on a line with their dominant arm outstretched at 90 degrees shoulder flexion, holding a pen. Paper is fixed to the wall at the side of the participant who touches the wall laterally to make a mark on the paper at the starting position. The participant is then instructed to reach as far forwards as possible without moving their feet or losing balance. A second mark on the paper is made at this point. The reach is measured as the mean distance reached on three attempts. Correlations between the PRT in standing and laboratory tests of limits of stability is moderate-to-high (r = 0.42 to 0.77). For children with CP, test-retest reliability and interrater reliability range from intraclass correlation coefficients (ICC) of 0.54 to 0.88 and 0.50 to 0.93, respectively [63,82].

2.2 **Activity and participation outcomes**

2.2.1 **Goal attainment:***Canadian Occupational Performance Measure (COPM)*

The COPM [56,83,84] identifies concerns regarding occupational performance and documents changes post BoNT-A rehabilitation [29] and will be a primary outcome measure in this study. It has demonstrated high re-test reliability (ICC 0.76-0.89), sensitivity to change and good content, construct and criterion validity for children with CP receiving BoNT-A [17,85-87]. The COPM will be administered using a semi-structured interview in collaboration with the caregiver/s and participant (dependent on age, cognitive ability and motivation to contribute) [88]. The child-adapted model has three sections: self-care (personal care, functional mobility and community management), productivity (play/school) and leisure (quiet recreation, active recreation and socialisation). Caregivers will be asked to identify three daily activities of concern where they or their child hope to improve after lower limb BoNT-A injections [17,85]. They will rate their perception of their child’s performance and their satisfaction with this performance on a 1–10 ordinal scale. A score change of two or more points is considered clinically significant [56].

2.2.2 **Efficiency of gait:***The One Minute Fast Walk Test (1MFWT)*

The 1MFWT is considered a good discriminator of functional ability for dynamic balance, muscle performance and endurance [60]. The test involves a five minute rest, followed by walking for one minute around a 20 metre oval track at maximum walking speed without running. Children are able use normal walking aids and wear orthoses. Distance is calculated to the nearest metre. The 1MFWT shows concurrent validity with the GMFM with a significant correlation between GMFM-88 score and distance walked (r = 0.92) [60]. Reliability has been established (ICC = 0.97, Standard Error of Measurement, SEM =4.0 m (4.1%)) with a score change of <17% (mean 1.28 m/s, SD 0.42) considered clinically meaningful [46].

2.2.3 **Gross motor ability:***Gross Motor Function Measure (GMFM-88)*

The GMFM-88 is a criterion-referenced measure designed for children with CP aged 0–18 years to assess motor function in five areas [61,62]. Items D (standing) and E (walking, running and jumping) will be administered as study participants are ambulant with goals frequently reflected in these areas. The GMFM-88 has good intra-rater (ICCs 0.92-0.99) and interrater (ICCs 0.87-0.99) reliability [61] and demonstrated validity to reflect change in gross motor function over time (ICCs 0.66-0.79) [89]. A ceiling and floor effect has been reported which highlights caution when interpreting results [61,90]. A change score of 1.3 points (total score), 1.2 points (Dimension D) and 1.6 points (Dimension E) is considered clinically meaningful [91,92]. A change score of 3.99 points further separates a great improvement from moderate or no improvement [91].

2.3 **Contextual**

2.3.1 **Quality of life:***The Cerebral Palsy Quality of Life Questionnaire (CPQOL-Child and CPQOL-Teen)*

The CP QOL-Child [64-66] and the CP QOL-Teen [67] are quality of life assessments designed for children and adolescents with CP aged four to 12 years and 13 to 18 years respectively. Both quantify well-being across seven key quality of life domains relevant to age group. Items are scored on a nine point rating scale, then summed and averaged to generate seven domain scores. The primary caregiver proxy versions will be used in this study as it is anticipated that the majority of children will be less than nine years of age. The CPQOL-Child and -Teen (primary caregiver proxy versions) have demonstrated good internal consistency (−Child: ICC 0.74-0.92; -Teen: Cronbach’s alphas 0.81-0.96), test–retest reliability (−Child: ICC 0.76-0.89; -Teen: ICC 0.29-0.83) and adequate construct validity supported by the pattern of correlations with scales including the Child Health Questionnaire (−Child only), Pediatric Quality of Life Inventory (−Teen only), KIDSCREEN and GMFCS [66,67].

2.3.2 **Qualitative evaluation**

i.) **Caregiver, child and physiotherapist satisfaction**

Post intervention (T2), custom-designed questionnaires will be used to evaluate satisfaction with the physiotherapy rehabilitation format. Questions will be open-ended and answers reviewed by one co-investigator (RT) to determine consistent themes.

ii.) **Child’s motivation and engagement with the physiotherapy intervention**

To evaluate whether there is a relationship between study outcomes and participant’s performance in therapy sessions, treating physiotherapists will be asked to rate each participant’s motivation, engagement and compliance with the physiotherapy exercises after each session using custom-designed five point Likert Scales.

iii.) **Child’s ongoing activity levels and maintenance of goals**

At six months post BoNT-A (T3), each caregiver will complete a custom-designed questionnaire to report their child’s access to ongoing therapy, completion of HEP, structured and un-structured activities, as well as caregiver’s perception of maintenance of goal areas on a ten point Likert Scale.

3. **Adverse events**

Treating physiotherapists will record any adverse events following each physiotherapy session. Adverse events will be classified as: *Mild:* awareness of sign or symptom, but easily tolerated; *Moderate:* discomfort enough to cause interference with usual activity; *Severe*: incapacitating with inability to do usual activity. Standard reporting and follow-up will be adhered to as per current Queensland Health protocol.

### Analyses

Statistical analysis will be undertaken by an investigator masked to group allocation (LS). Primary analysis will use the intention to treat principle, using the last observation carried forward for participants who withdraw before the end of the trial. Baseline data from each outcome measure for each treatment group will be reported using descriptive statistics. Continuous data will be compared between groups by fitting a regression model using generalized estimating equations [93] to baseline, 10 and 26 week measurements with an interaction term between the intervention group and a three-level factor indicating time of measurement. The generalized estimating equation model will assume a Gaussian family, identity link, and unstructured working correlation matrix for repeated measurements on participants. Conventional variance estimates will be used. Where continuous data exhibit skewness not overcome by transformation, non-parametric methods (Mann–Whitney U) will be used for simple comparisons. Statistical significance will be at p < 0.05 and analyses will be performed using STATA 11.

## Discussion

This protocol paper presents the background and design of an assessor-masked, randomised comparison trial evaluating the efficacy of group versus individual models of physiotherapy following intramuscular lower limb BoNT-A injections for ambulant children with CP. To our knowledge, this will be the first study to compare these two physiotherapy models with this population using outcome measures across domains of the ICF. It will address the essential need for rehabilitation services to consider flexible models of service delivery in response to family preferences and an increasing emphasis on improving goal-directed functional activity performance and societal participation within a family-centred framework [94]. Importantly, children, caregiver/s and therapists will have a greater informed choice of post BoNT-A rehabilitation delivery options.

### Ethics

The research ethics boards at the Royal Children’s Hospital, Brisbane, Australia (HREC2008/089) and the Cerebral Palsy League, Brisbane, Australia (CPLQ2009/2010-1030) have granted approval for the study.

## Abbreviations

RT: Rachel Thomas; LJ: Leanne Johnston; RB: Roslyn Boyd; LS: Leanne Sakzewski; MK: Megan Kentish; T1: Assessment Time 1 (baseline); T2: Assessment Time 2 (10 weeks post BoNT-A injections, efficacy); T3: Assessment Time 3 (26 weeks post BoNT-A injections, retention); CP: Cerebral palsy; BoNT-A: Botulinum Toxin- Type A; GMFCS-E&R: Gross Motor Function Classification System- Expanded and Revised; ICF: International Classification of Functioning, Disability and Health; RCT: Randomised controlled trial; GMFM-88: Gross Motor Function Measure-88; EVGS: Edinburgh Visual Gait Score for Cerebral Palsy; 3DGA: Three dimensional gait analysis; COPM: Canadian Occupational Performance Measure; GAS: Goal Attainment Scale; NDT: Neurodevelopmental therapy; CPT: Conventional physiotherapy; CAPE: Children’s Assessment of Participation and Enjoyment; CP Health: Queensland Cerebral Palsy Health Service, Brisbane, Queensland; HEP: Home Exercise Programme; FMS: Functional Mobility Scale; QCGL: Queensland Children’s Gait Laboratory; PRT: Pediatric Reach Test; ICC: Intraclass correlation coefficient; 1MFWT: One Minute Fast Walk Test; SEM: Standard error of measurement; CI: Confidence Interval; CPQOL-Child: Cerebral Palsy Quality of Life Questionnaire for Children; CPQOL-Teen: Cerebral Palsy Quality of Life Questionnaire for adolescents; QOL: Quality of Life; SLS: Single leg stance.

## Competing interests

The authors declare that they have no competing interests.

## Authors’ contributions

RT, MK and LJ were responsible for the study concept, design and ethics applications. LS provided statistical advice for the study design. MK and LJ obtained funding for development of the study protocol and ethics submission. MK obtained funding for the study. RT registered the trial with ACTRN and drafted the manuscript which was critically reviewed by all authors. All authors read and approved the final manuscript.

## Pre-publication history

The pre-publication history for this paper can be accessed here:

http://www.biomedcentral.com/1471-2431/14/35/prepub
